# Supplementary figures and images for: Tissue-specific O-GlcNAcylation profiling identifies substrates in translational machinery in Drosophila mushroom body contributing to olfactory learning
Source: eLife. 2024 Apr 15;13:e91269. doi: 10.7554/eLife.91269 (PMC11018347; doi:10.7554/eLife.91269)

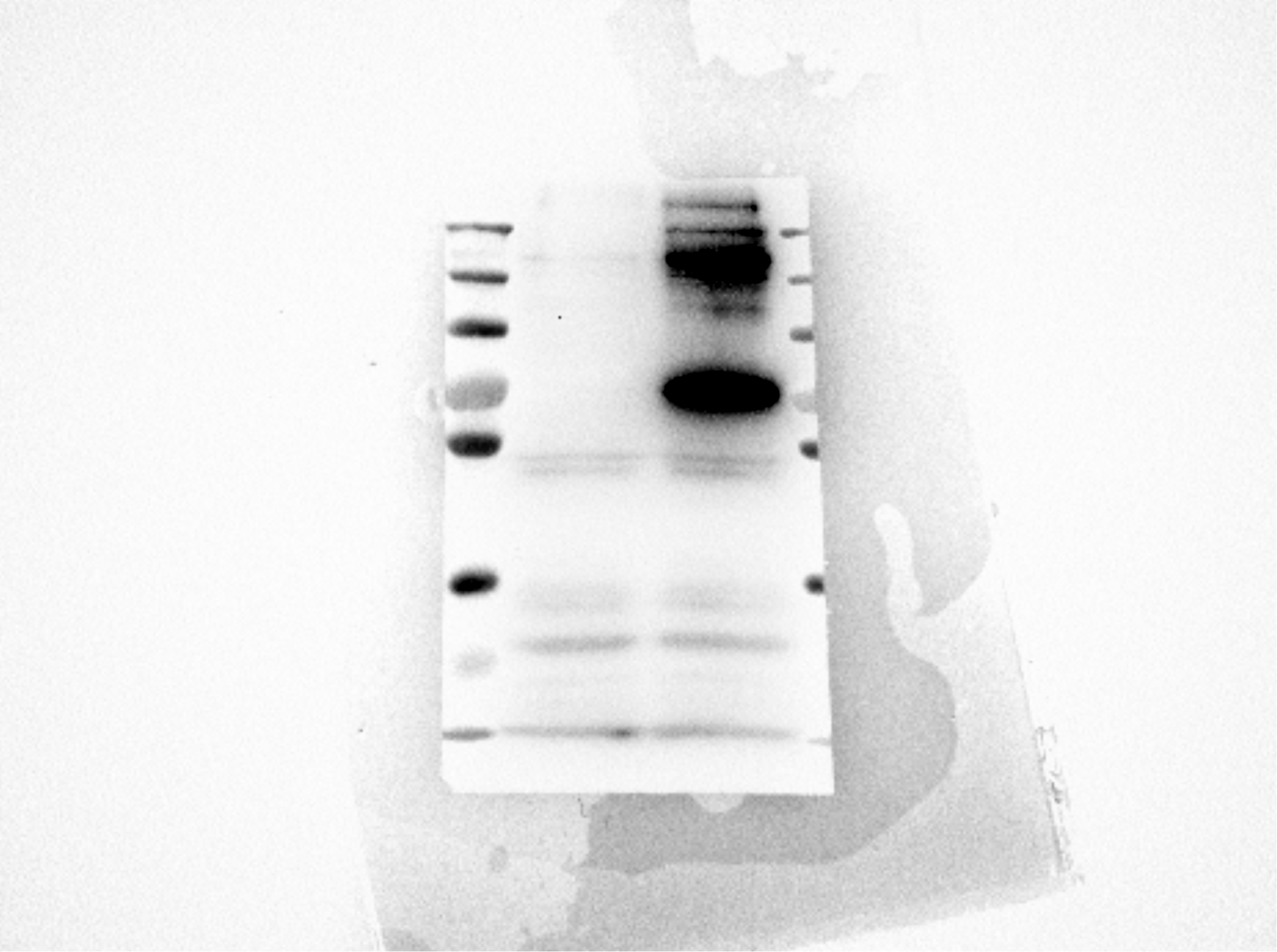

Supplement: Figure 2—source data 1. [file elife-91269-fig2-data1.zip › Figure_2-source_data_1/Figure_2-source_data_1_Figure_2C_IP RL2.tif]

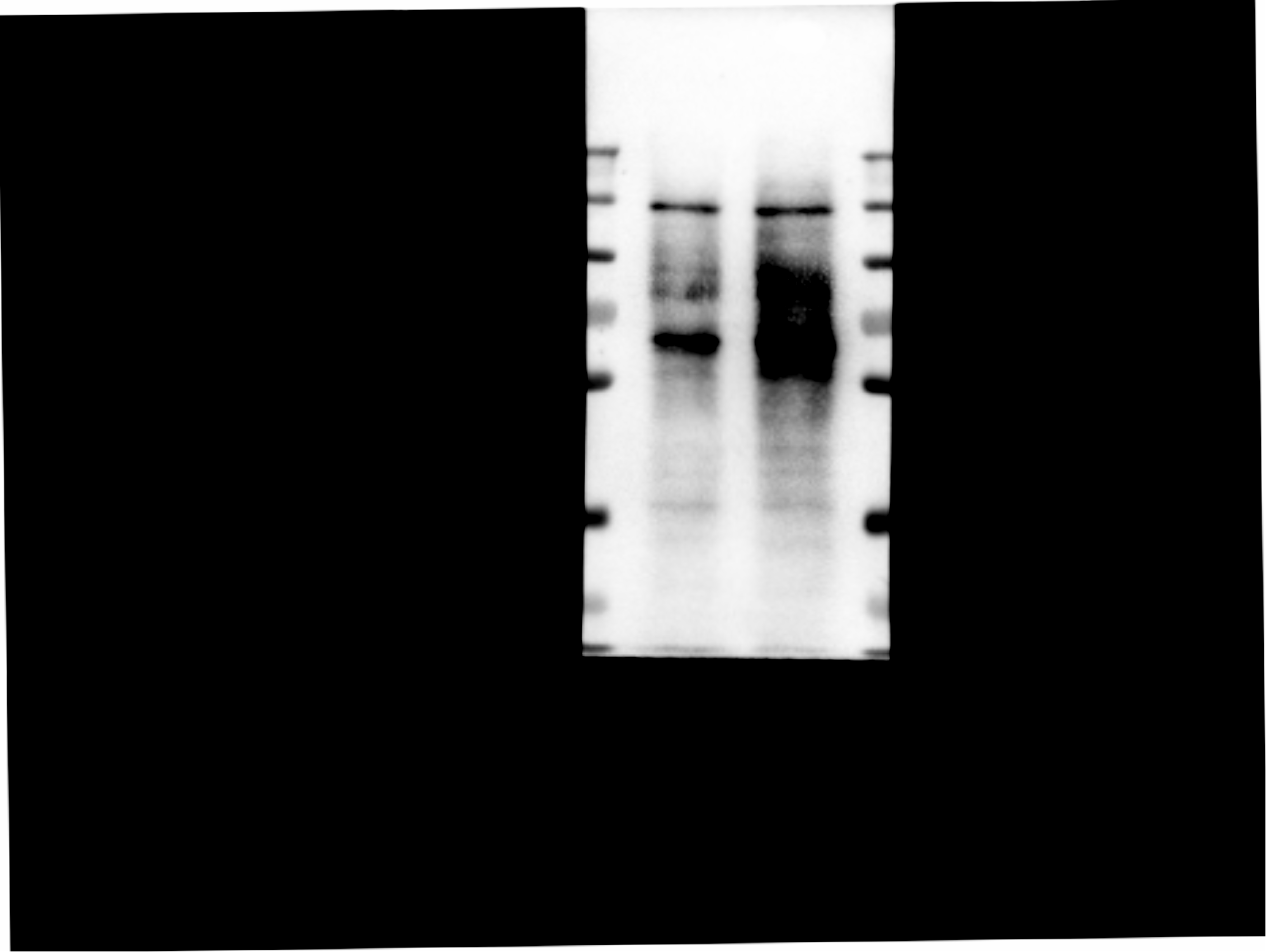

Supplement: Figure 2—source data 1. [file elife-91269-fig2-data1.zip › Figure_2-source_data_1/Figure_2-source_data_1_Figure_2C_IP Streptavidin.tif]

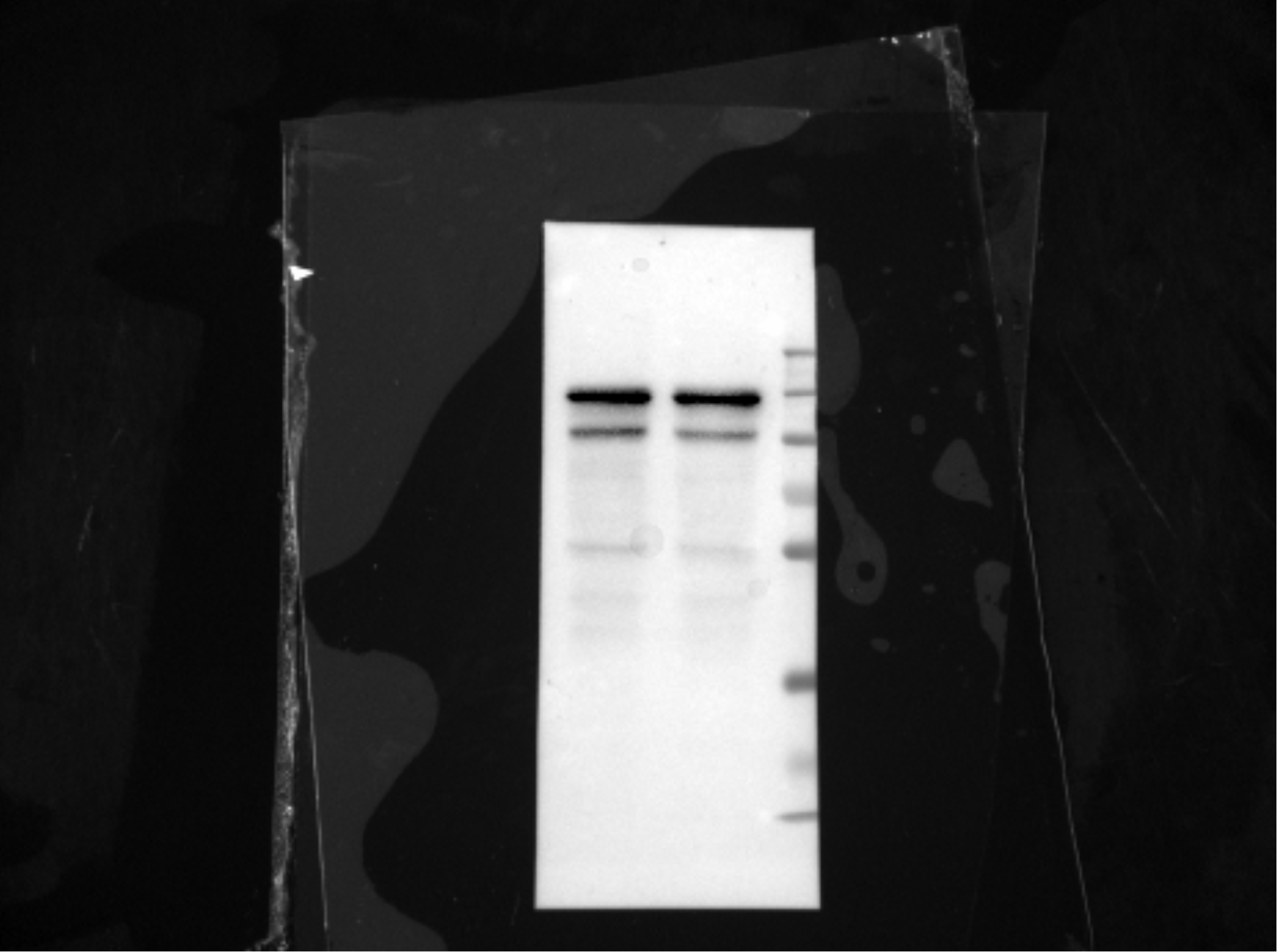

Supplement: Figure 2—source data 1. [file elife-91269-fig2-data1.zip › Figure_2-source_data_1/Figure_2-source_data_1_Figure_2C_Input HA.tif]

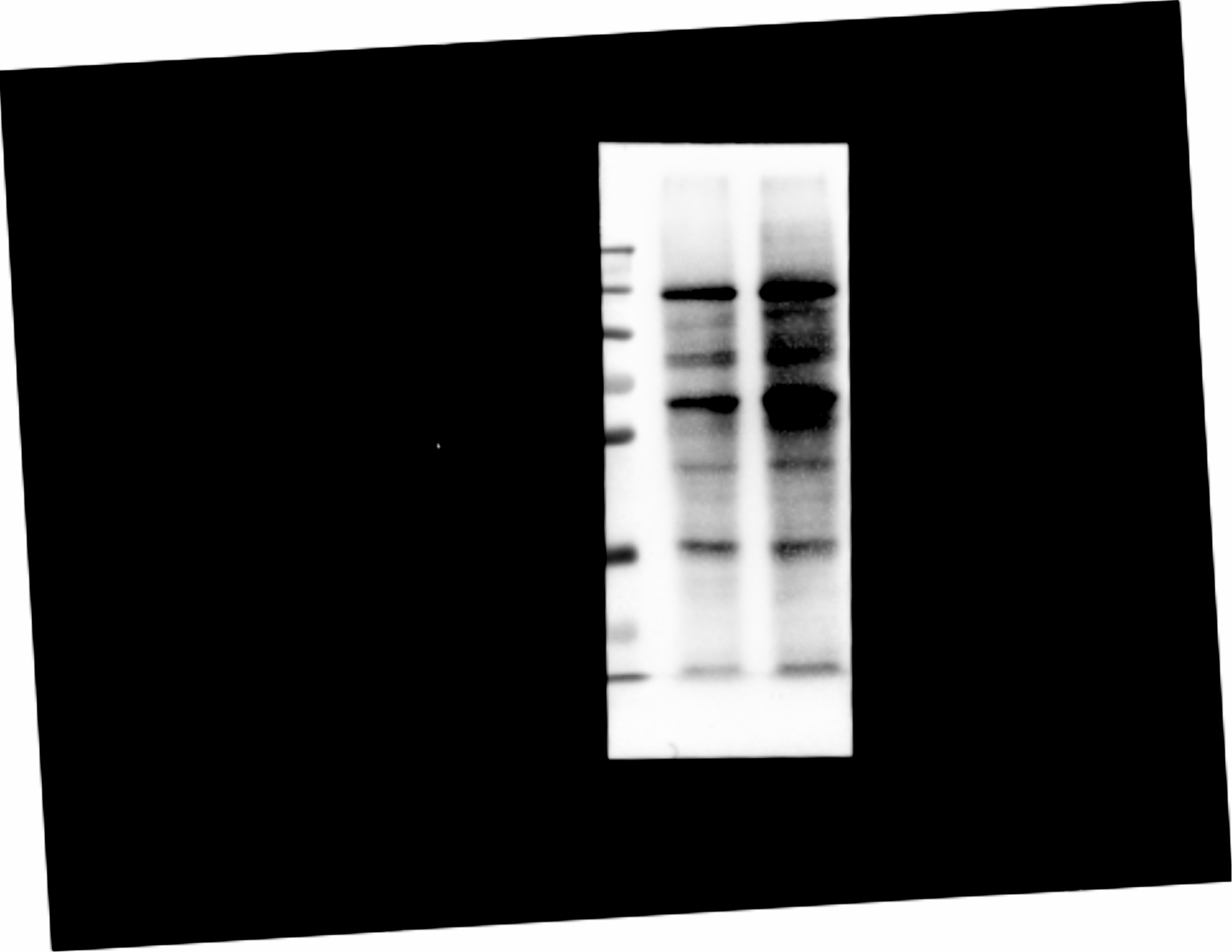

Supplement: Figure 2—source data 1. [file elife-91269-fig2-data1.zip › Figure_2-source_data_1/Figure_2-source_data_1_Figure_2C_Input Streptavidin.tif]

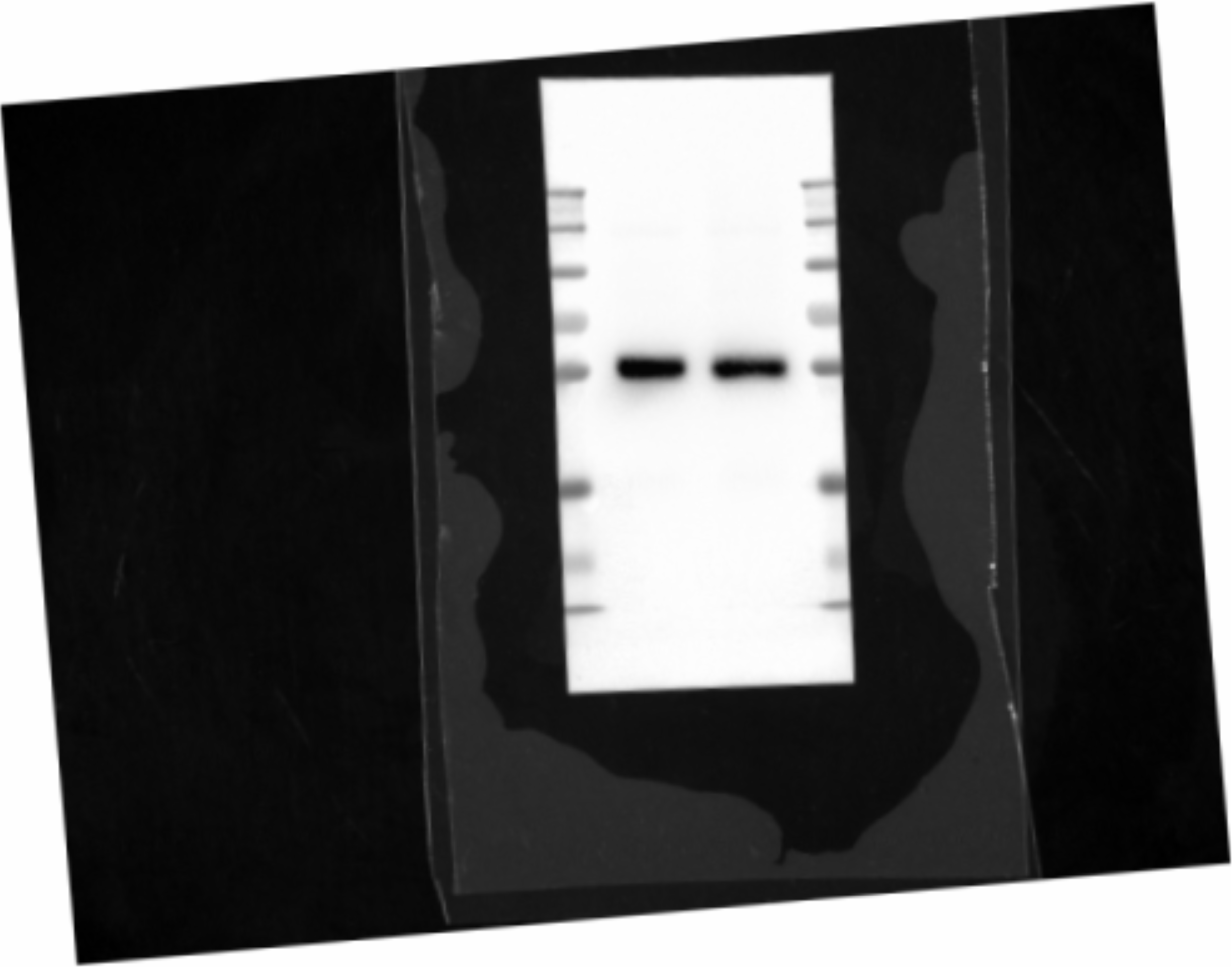

Supplement: Figure 2—source data 1. [file elife-91269-fig2-data1.zip › Figure_2-source_data_1/Figure_2-source_data_1_Figure_2C_Input Tubulin.tif]

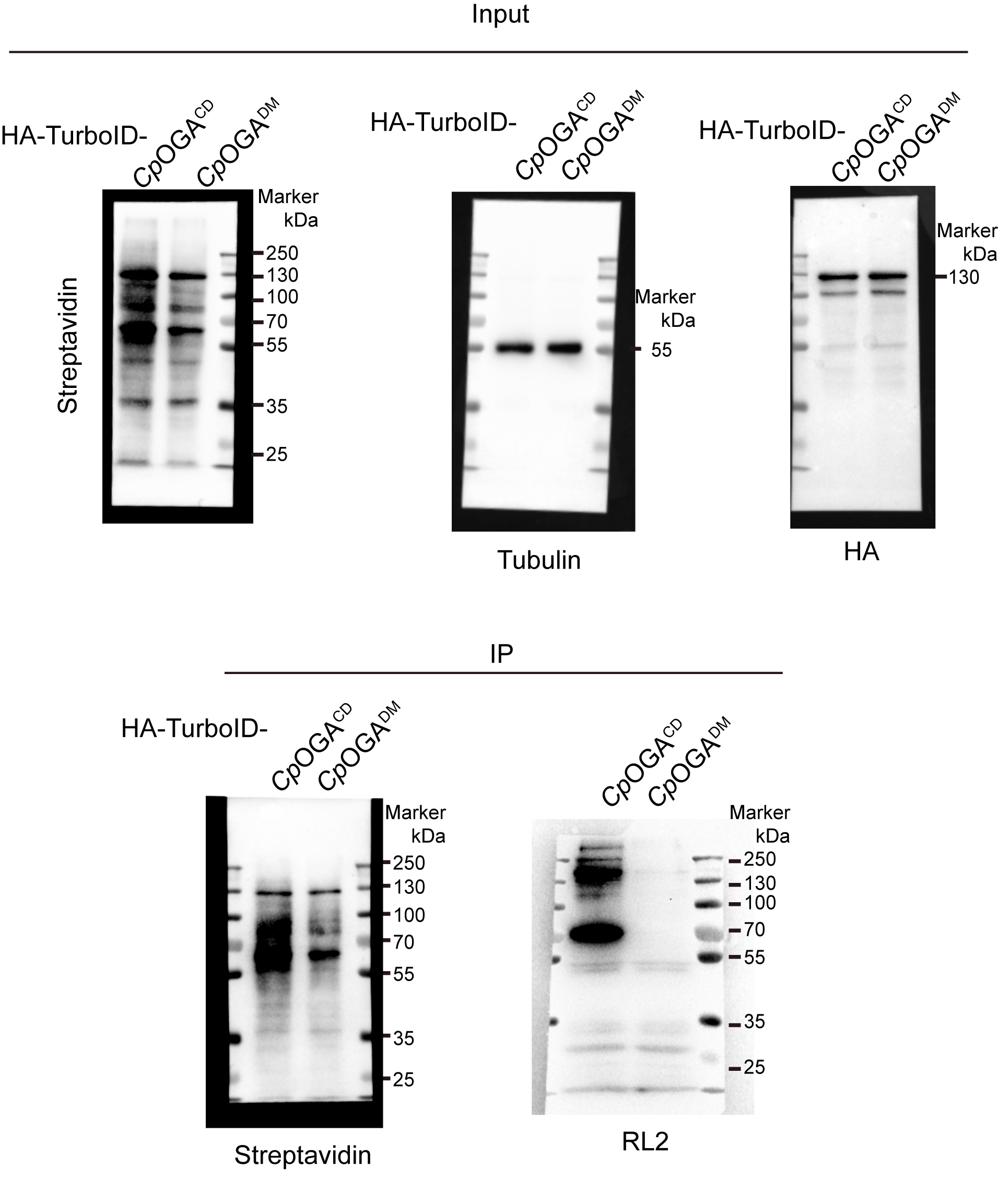

Supplement: Figure 2—source data 2. [file elife-91269-fig2-data2.zip › Figure_2-source_data_2/Figure_2-source_data_2-2C.tif]

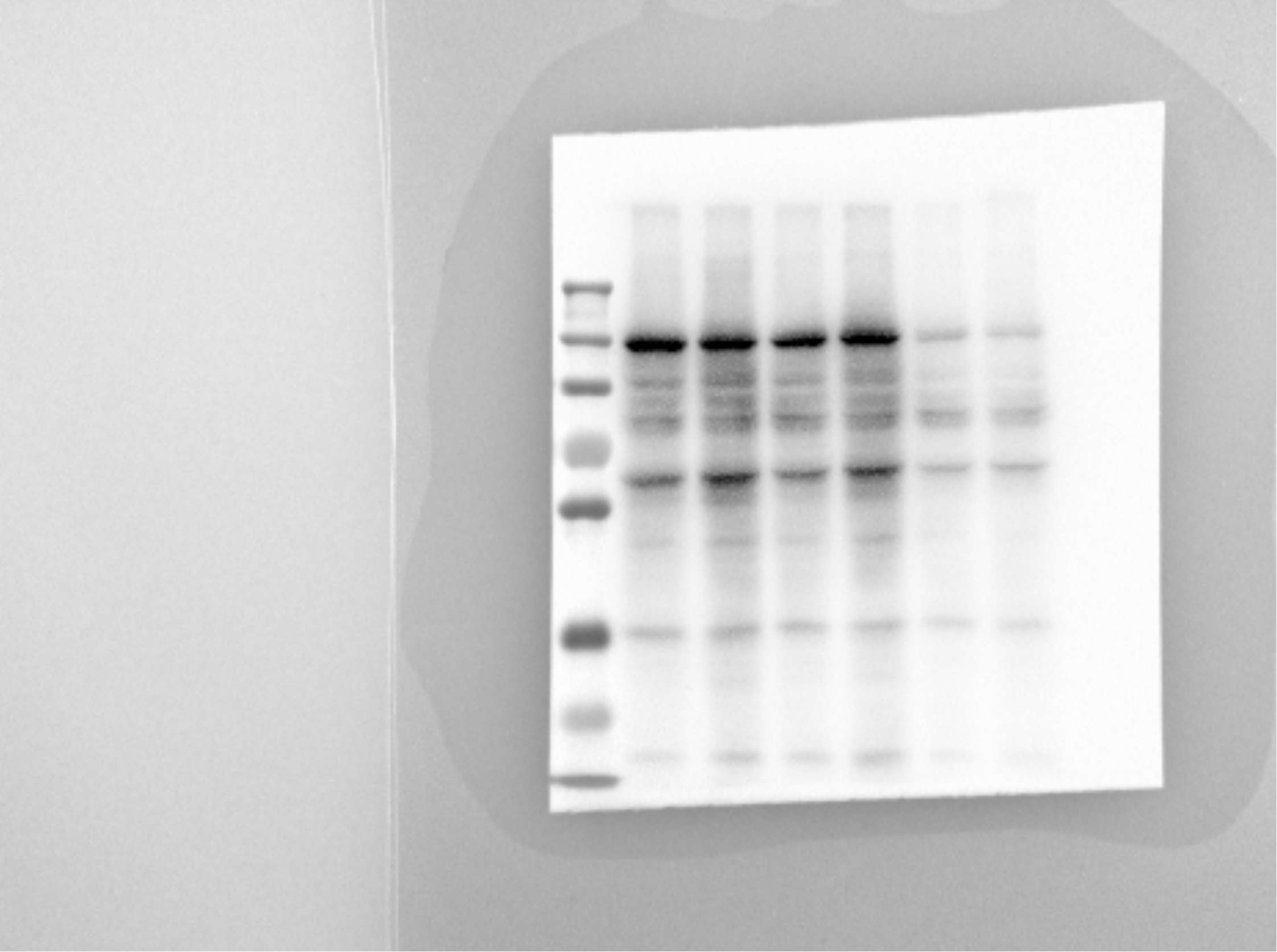

Supplement: Figure 2—figure supplement 1—source data 1. [file elife-91269-fig2-figsupp1-data1.zip › Figure 2-figure supplement 1-source_data_1/Figure 2-figure supplement 1-source_data_1A_Streptavidin.tif]

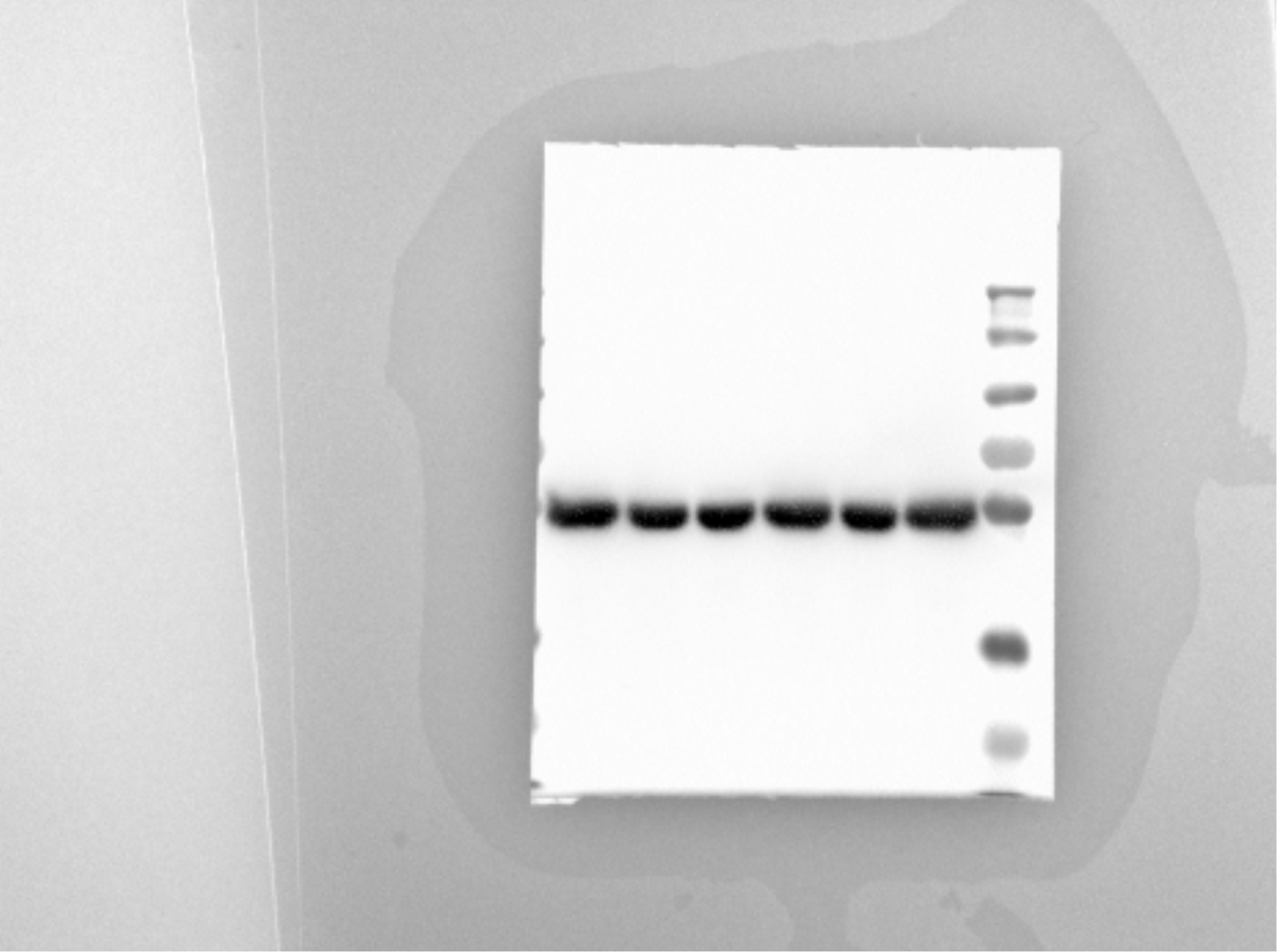

Supplement: Figure 2—figure supplement 1—source data 1. [file elife-91269-fig2-figsupp1-data1.zip › Figure 2-figure supplement 1-source_data_1/Figure 2-figure supplement 1-source_data_1A_Tubulin.tif]

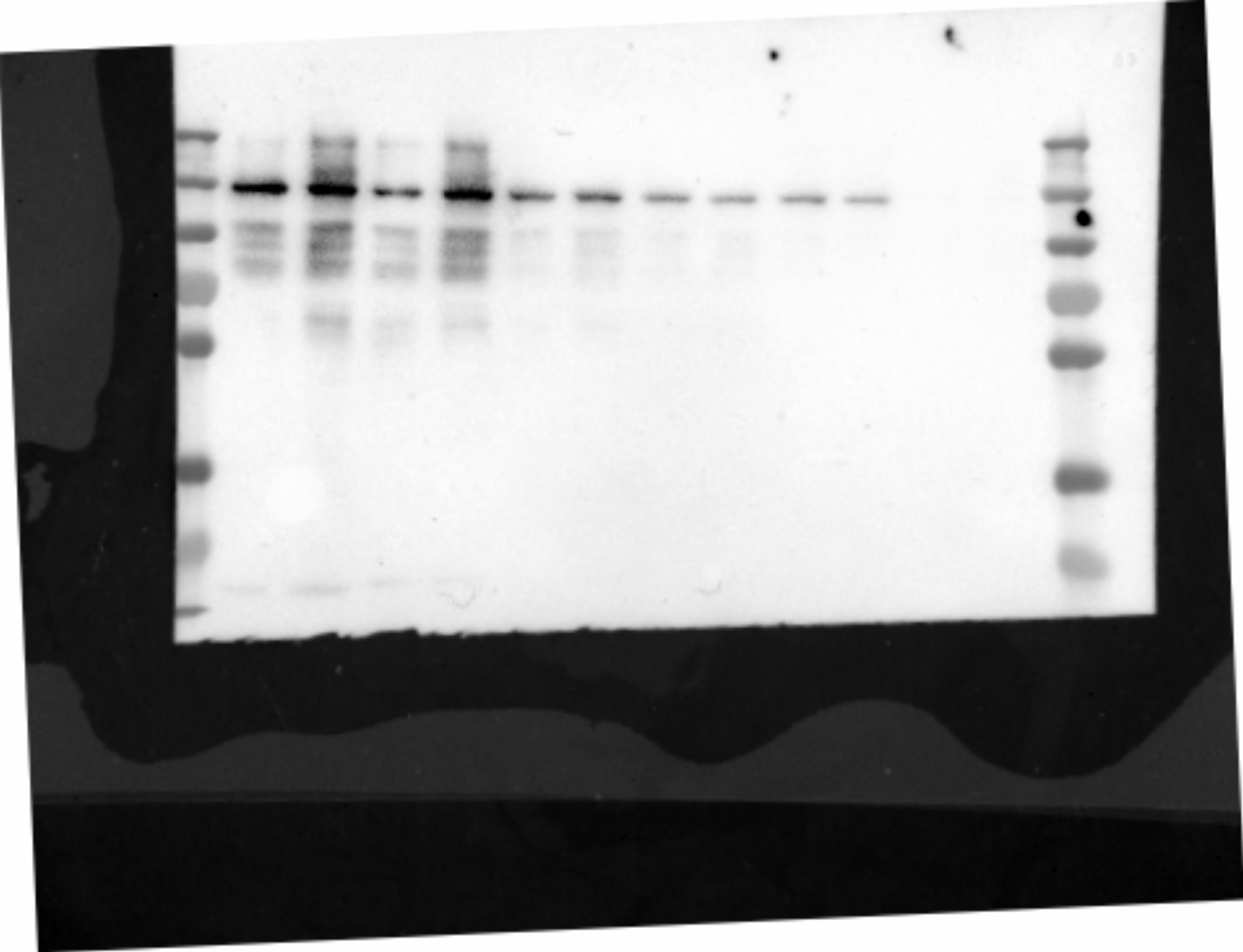

Supplement: Figure 2—figure supplement 1—source data 1. [file elife-91269-fig2-figsupp1-data1.zip › Figure 2-figure supplement 1-source_data_1/Figure 2-figure supplement 1-source_data_1B_Streptavidin.tif]

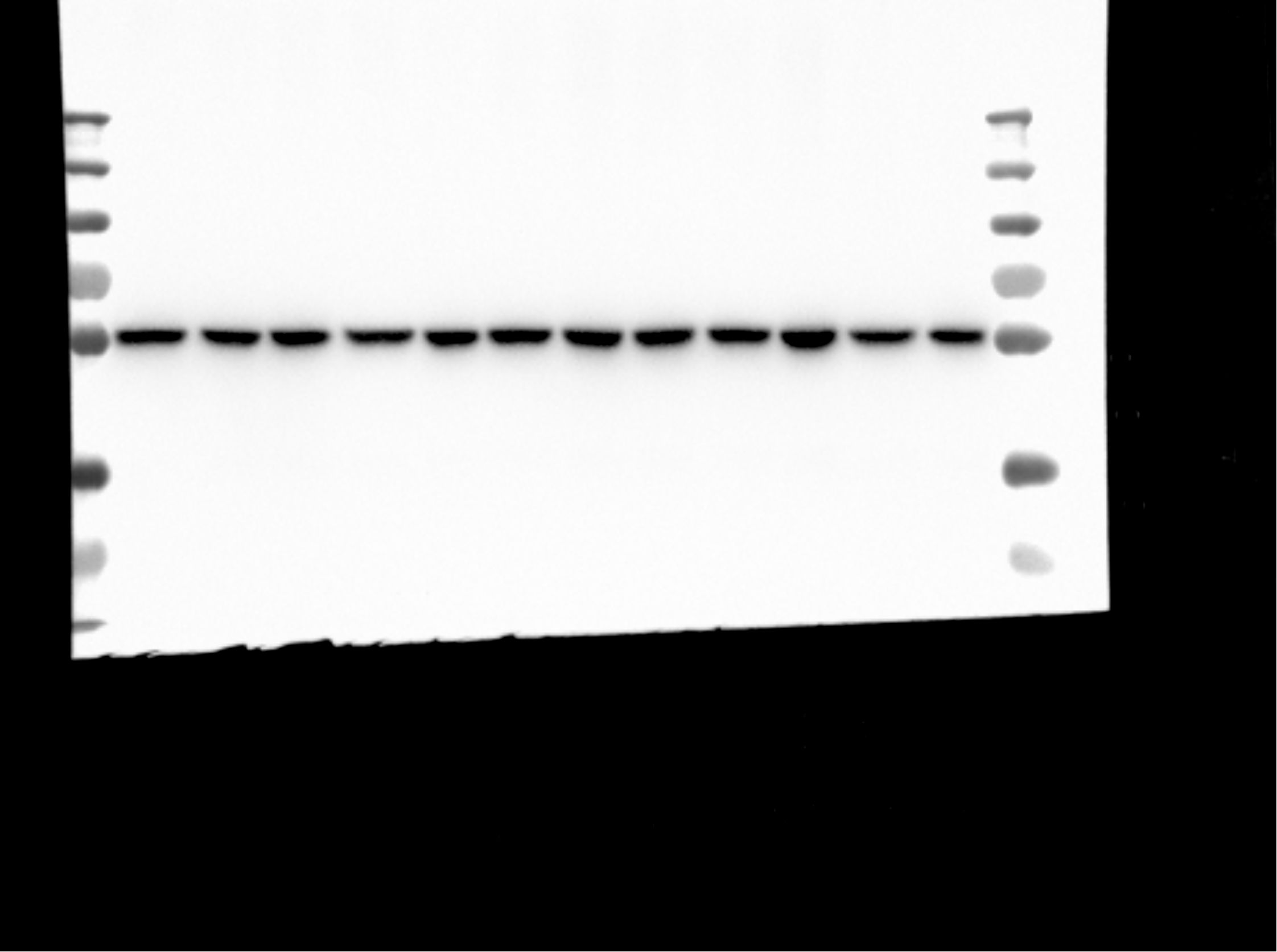

Supplement: Figure 2—figure supplement 1—source data 1. [file elife-91269-fig2-figsupp1-data1.zip › Figure 2-figure supplement 1-source_data_1/Figure 2-figure supplement 1-source_data_1B_Tubulin.tif]

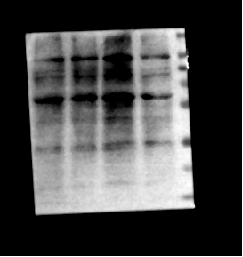

Supplement: Figure 2—figure supplement 1—source data 1. [file elife-91269-fig2-figsupp1-data1.zip › Figure 2-figure supplement 1-source_data_1/Figure 2-figure supplement 1-source_data_1C_RL2.tif]

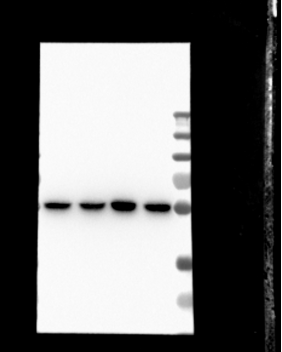

Supplement: Figure 2—figure supplement 1—source data 1. [file elife-91269-fig2-figsupp1-data1.zip › Figure 2-figure supplement 1-source_data_1/Figure 2-figure supplement 1-source_data_1C_Tubulin.tif]

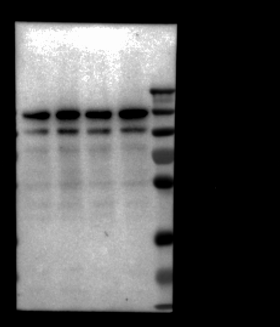

Supplement: Figure 2—figure supplement 1—source data 1. [file elife-91269-fig2-figsupp1-data1.zip › Figure 2-figure supplement 1-source_data_1/Figure 2-figure supplement 1-source_data_1D_HA.tif]

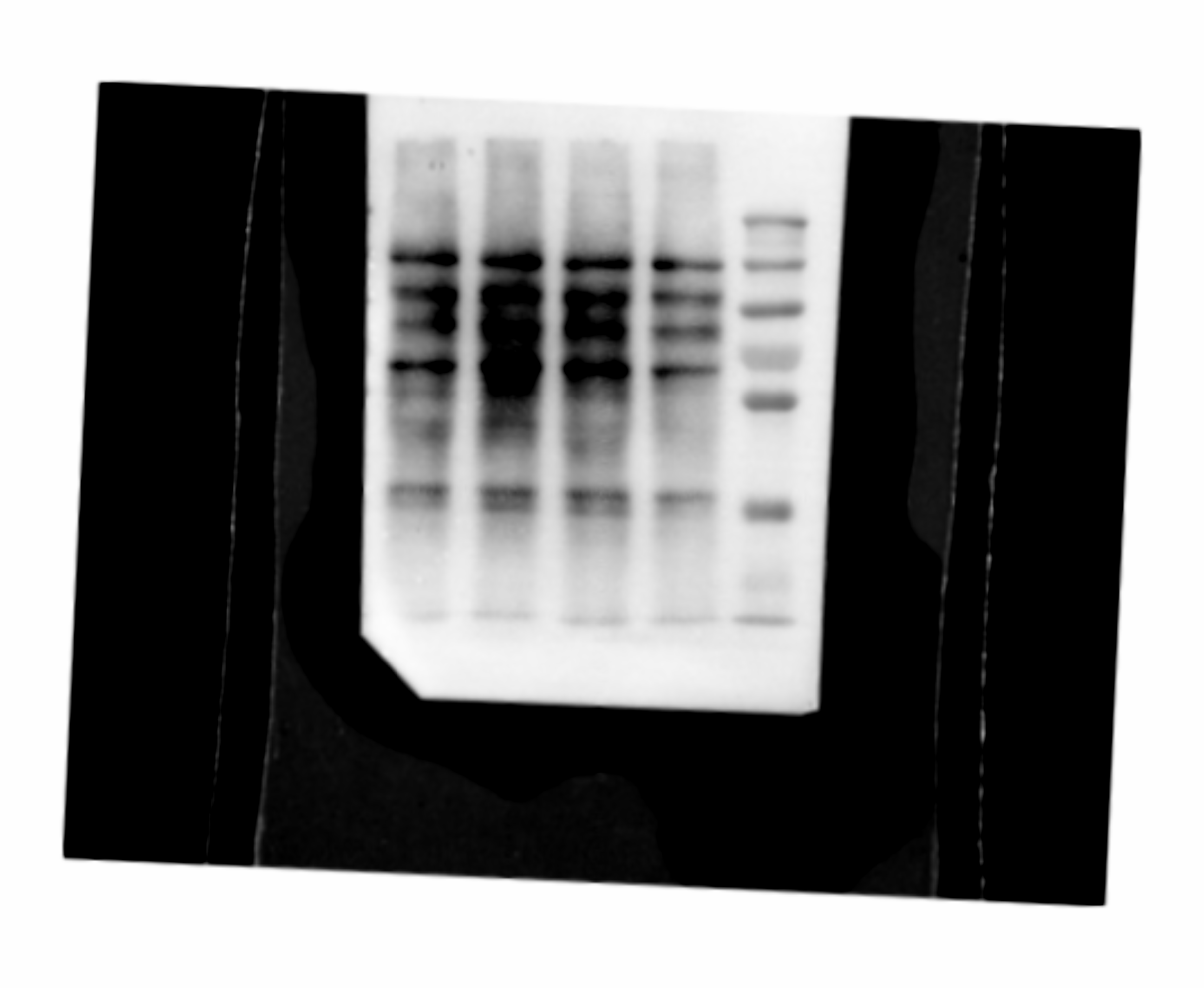

Supplement: Figure 2—figure supplement 1—source data 1. [file elife-91269-fig2-figsupp1-data1.zip › Figure 2-figure supplement 1-source_data_1/Figure 2-figure supplement 1-source_data_1D_Streptavidin.tif]

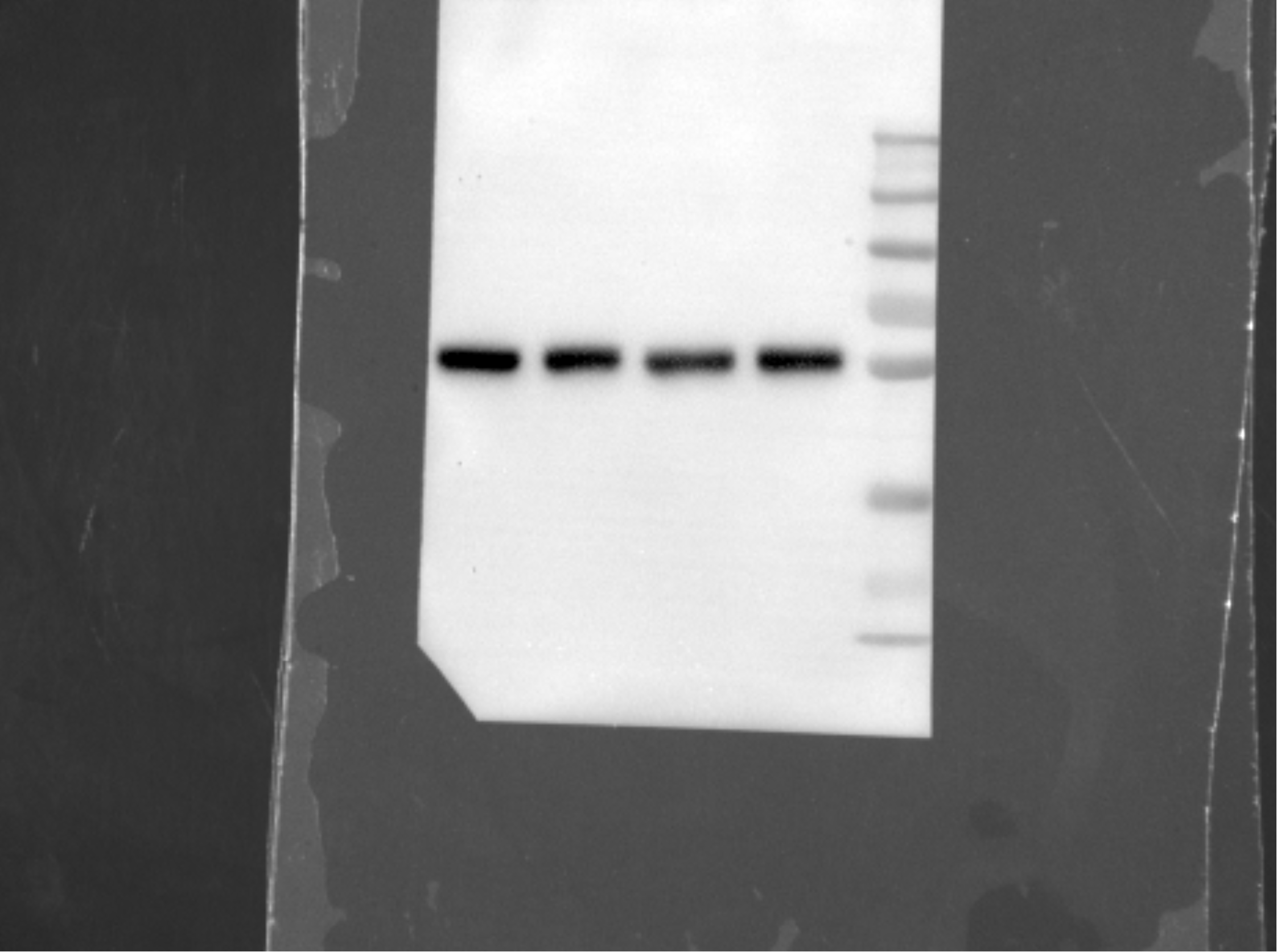

Supplement: Figure 2—figure supplement 1—source data 1. [file elife-91269-fig2-figsupp1-data1.zip › Figure 2-figure supplement 1-source_data_1/Figure 2-figure supplement 1-source_data_1D_Tubulin.tif]

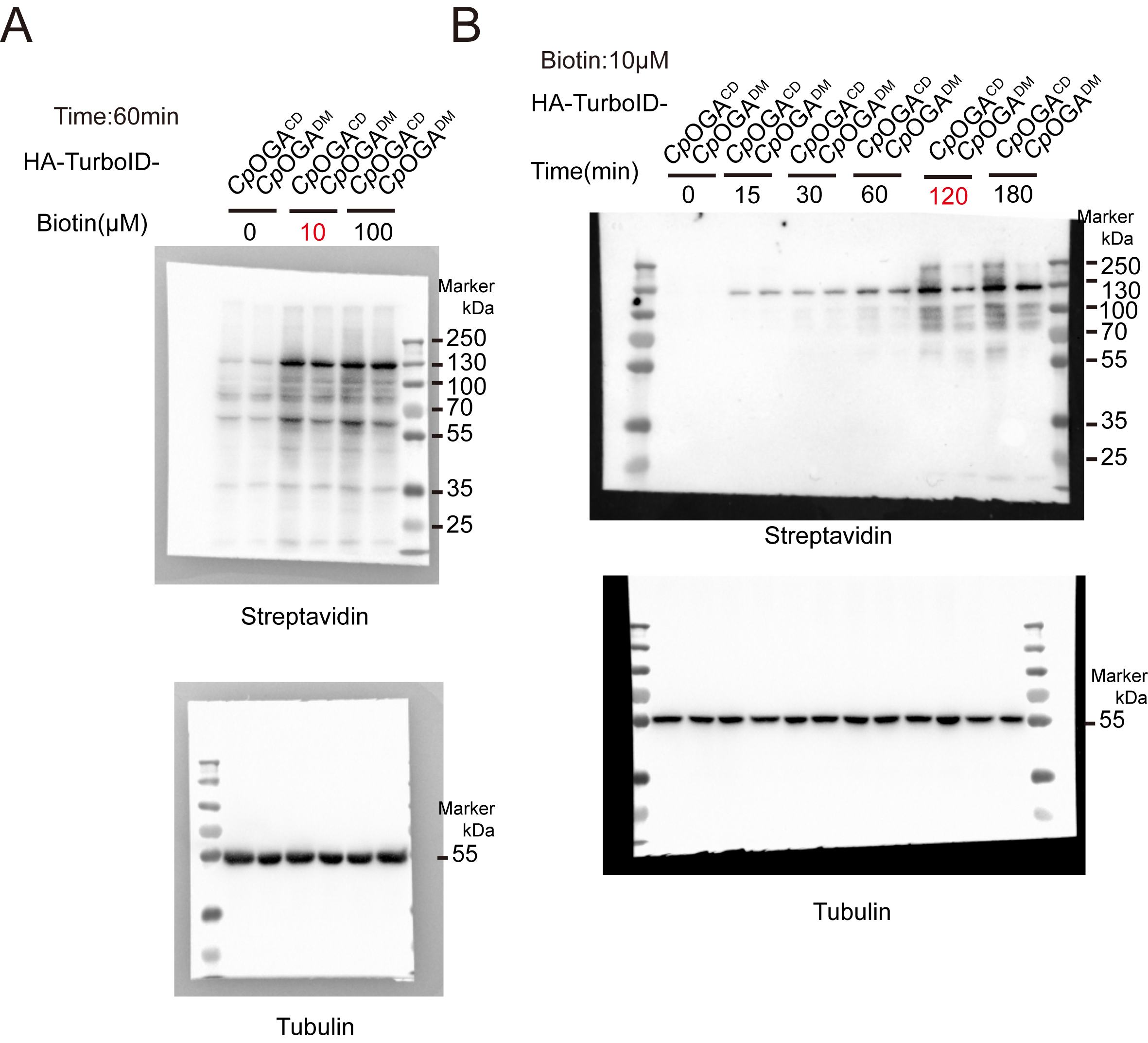

Supplement: Figure 2—figure supplement 1—source data 2. [file elife-91269-fig2-figsupp1-data2.zip › Figure 2-figure supplement 1-source_data_2/Figure 2-figure supplement 1-source_data_2A B.tif]

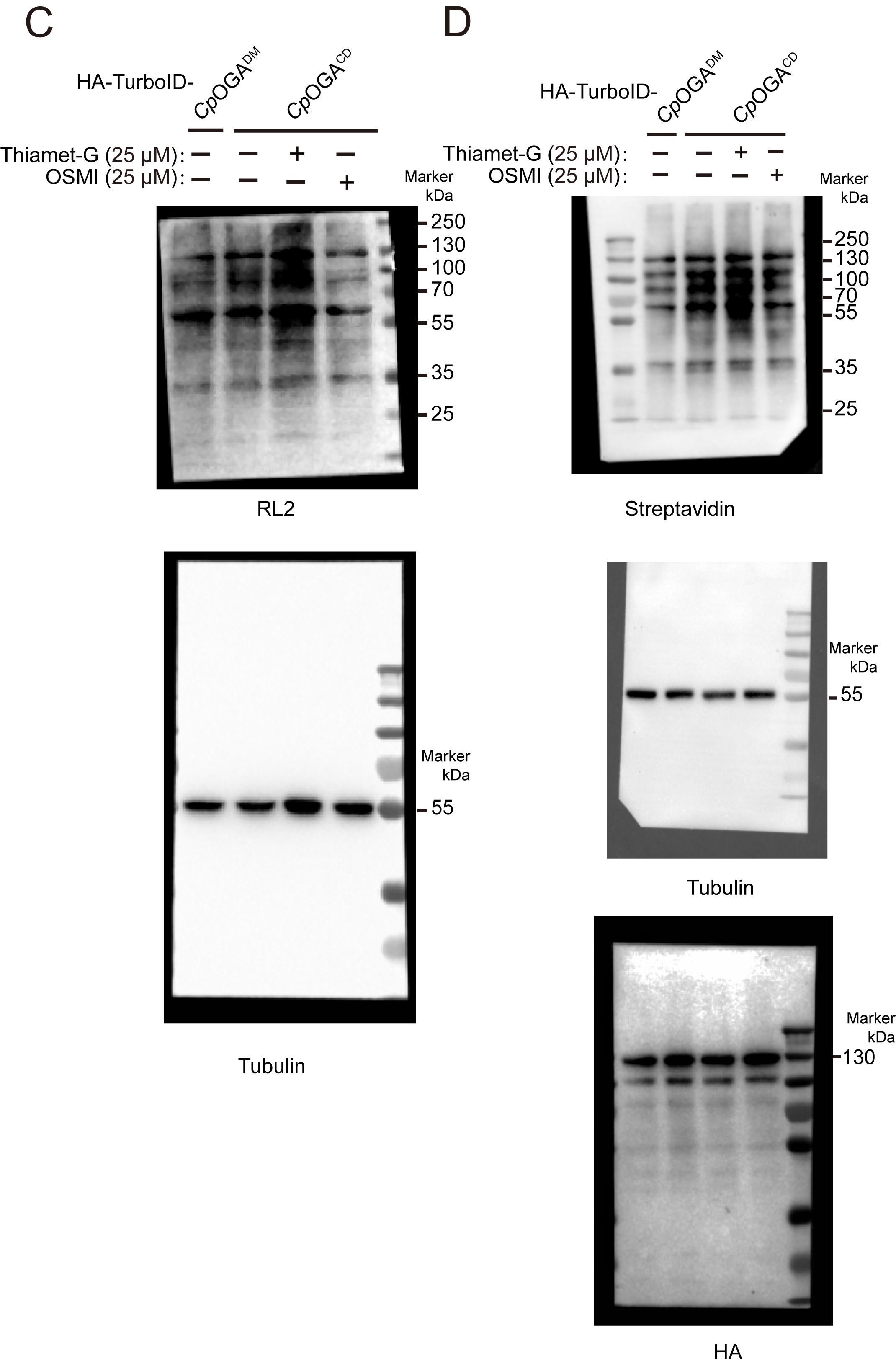

Supplement: Figure 2—figure supplement 1—source data 2. [file elife-91269-fig2-figsupp1-data2.zip › Figure 2-figure supplement 1-source_data_2/Figure 2-figure supplement 1-source_data_2C D.tif]

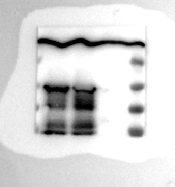

Supplement: Figure 3—source data 1. [file elife-91269-fig3-data1.zip › Figure_3-source_data_1/Figure_3-source_data_1_Figure_3B_INPUT HA.tif]

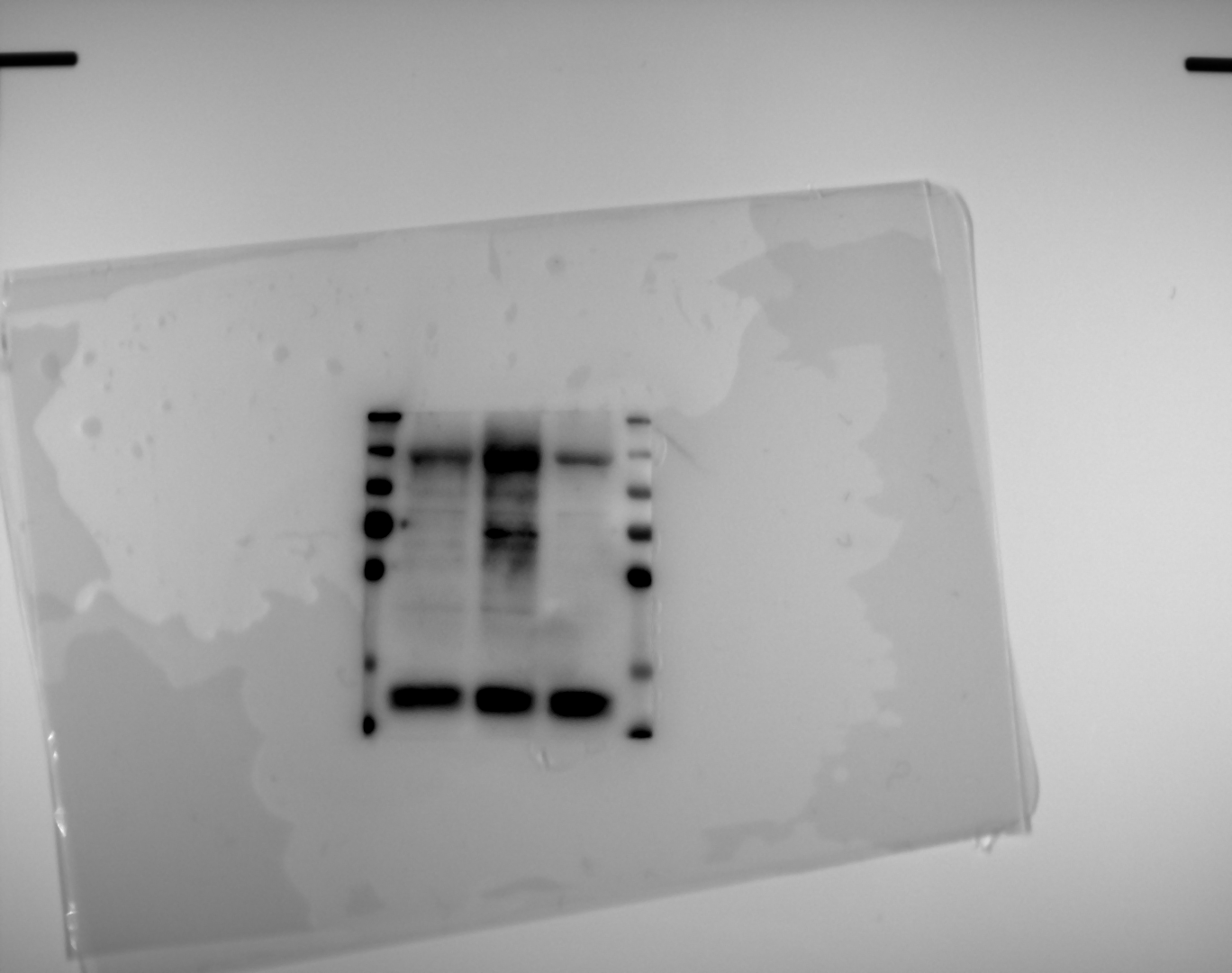

Supplement: Figure 3—source data 1. [file elife-91269-fig3-data1.zip › Figure_3-source_data_1/Figure_3-source_data_1_Figure_3B_IP RL2.tif]

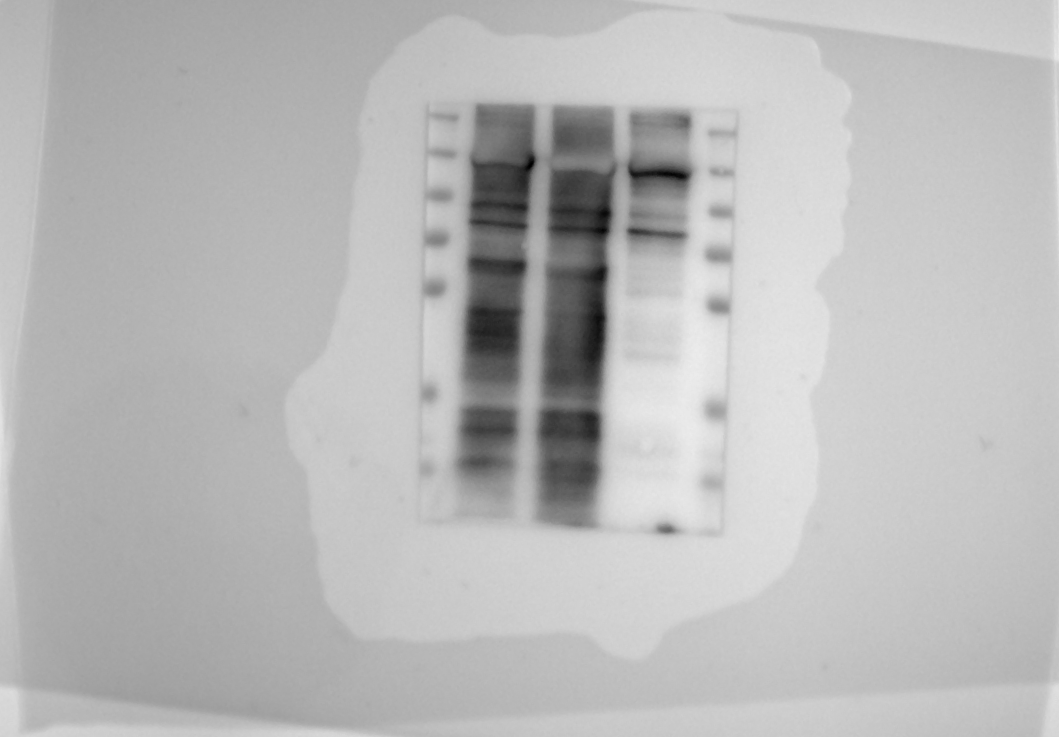

Supplement: Figure 3—source data 1. [file elife-91269-fig3-data1.zip › Figure_3-source_data_1/Figure_3-source_data_1_Figure_3B_IP Streptavidin.tif]

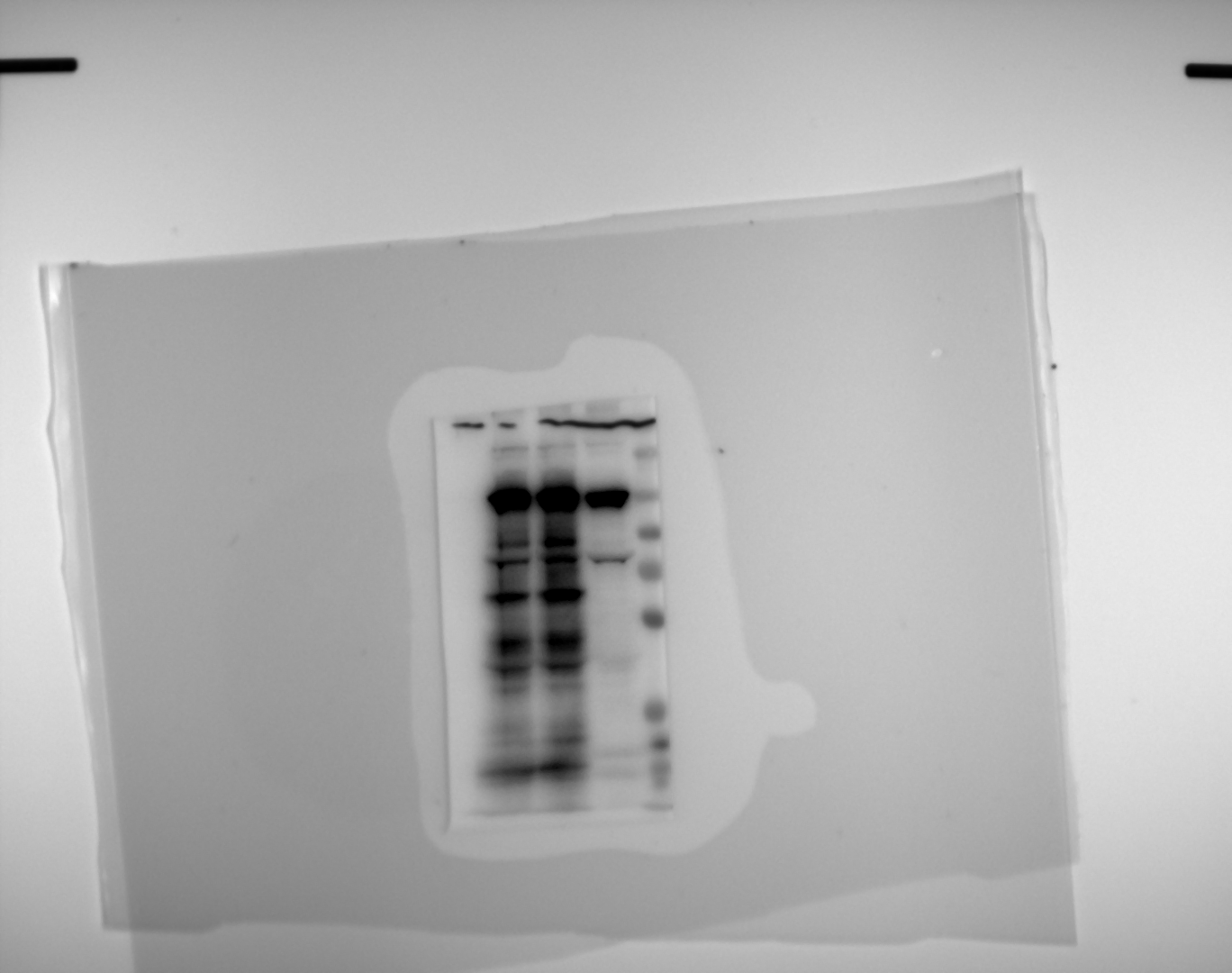

Supplement: Figure 3—source data 1. [file elife-91269-fig3-data1.zip › Figure_3-source_data_1/Figure_3-source_data_1_Figure_3B_Input Streptavidin.tif]

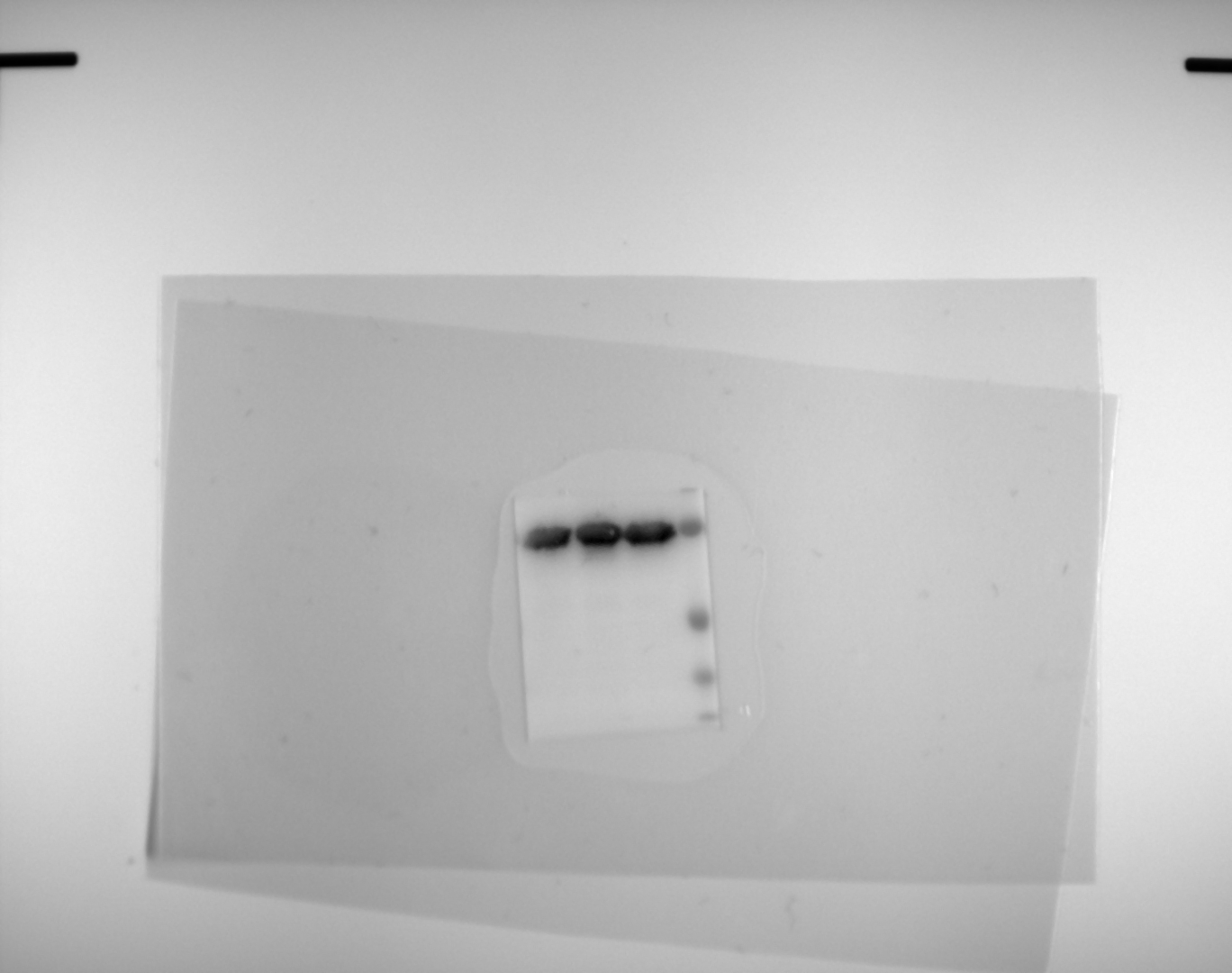

Supplement: Figure 3—source data 1. [file elife-91269-fig3-data1.zip › Figure_3-source_data_1/Figure_3-source_data_1_Figure_3B_Input Tubulin.tif]

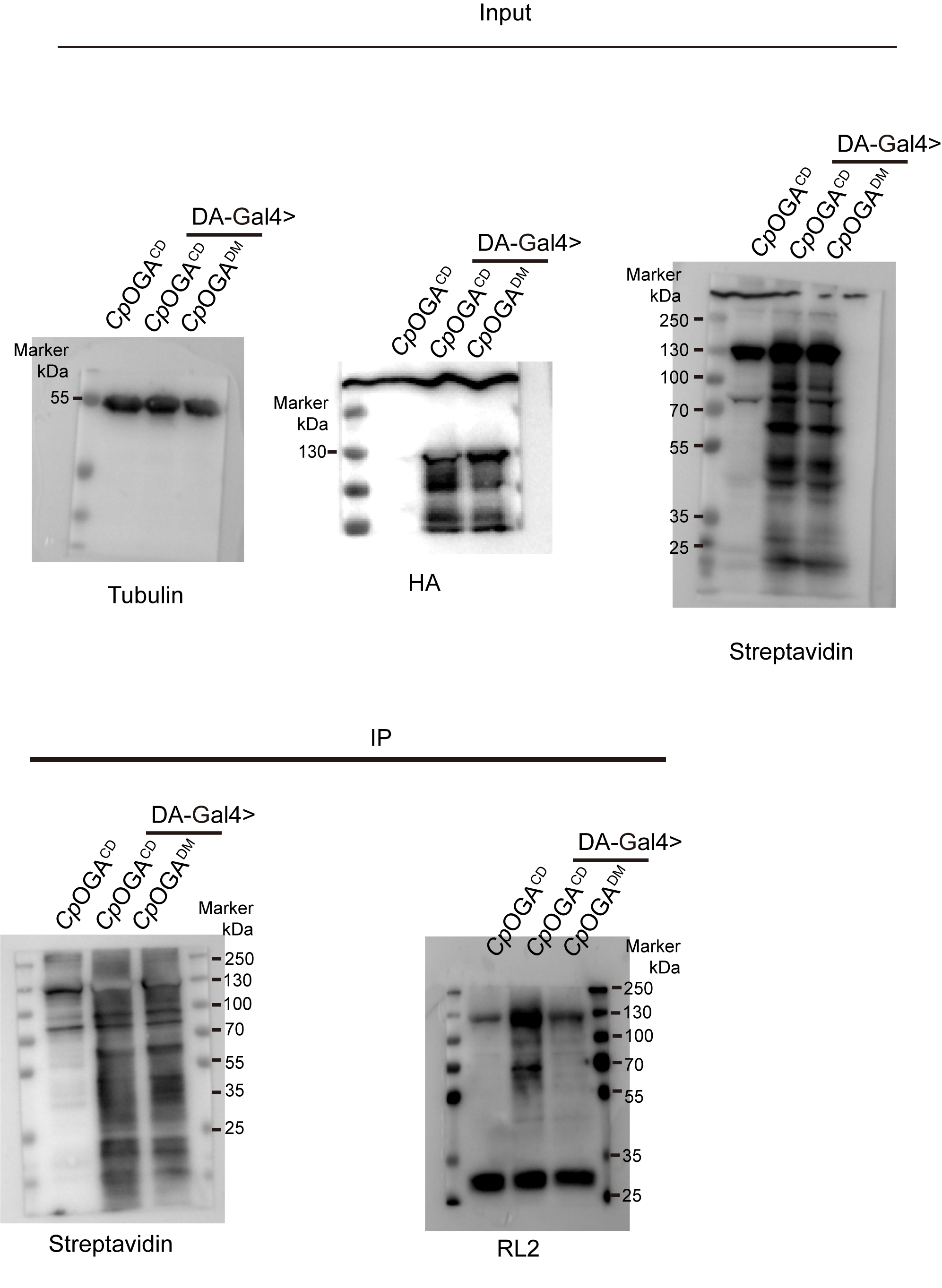

Supplement: Figure 3—source data 2. [file elife-91269-fig3-data2.zip › Figure_3-source_data_2/Figure_3-source_data_2-3B.tif]

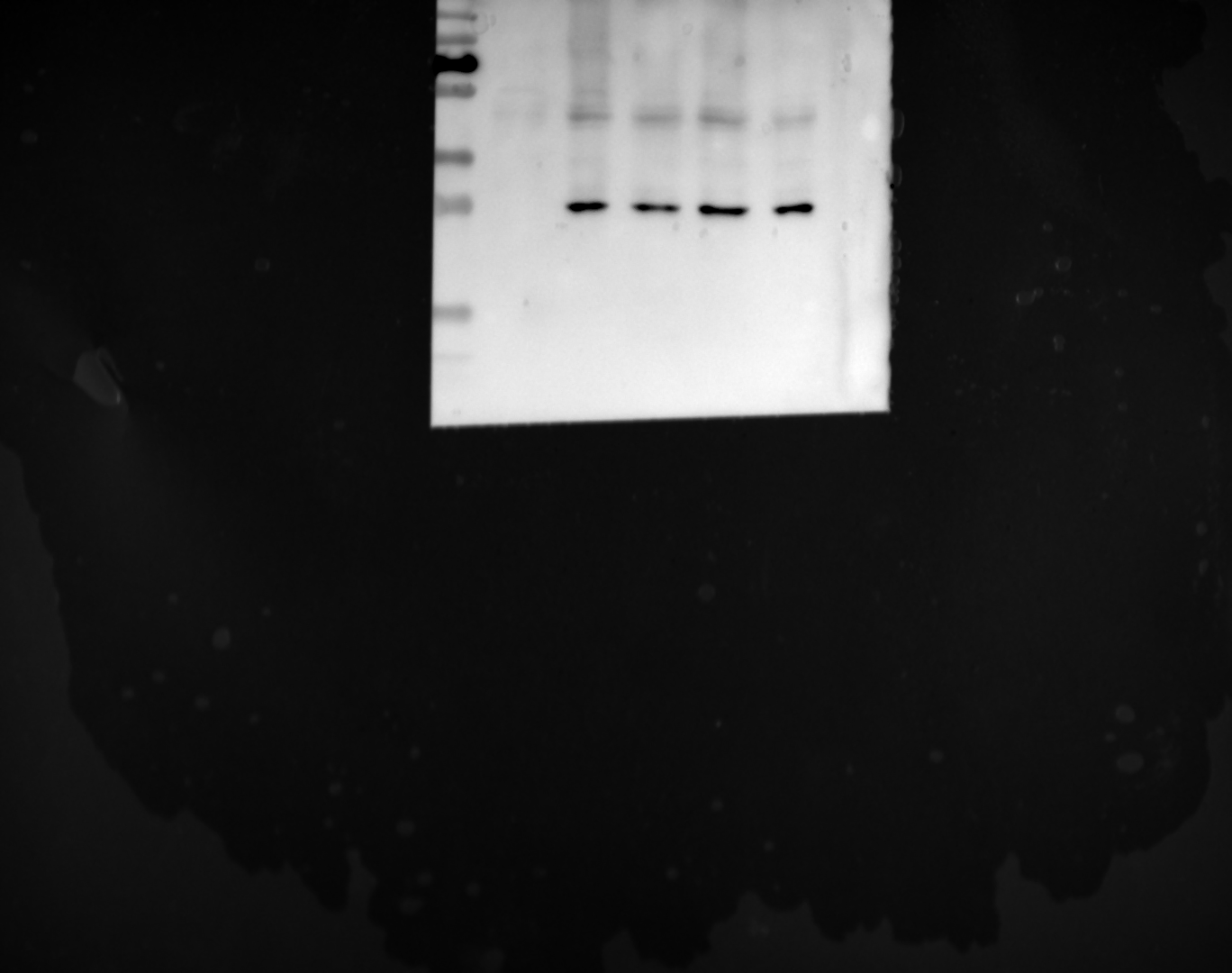

Supplement: Figure 4—source data 1. [file elife-91269-fig4-data1.zip › Figure_4-source_data_1/Figure_4-source_data_1_Figure_4B_IP Flag.tif]

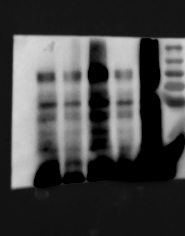

Supplement: Figure 4—source data 1. [file elife-91269-fig4-data1.zip › Figure_4-source_data_1/Figure_4-source_data_1_Figure_4B_IP RL2.tif]

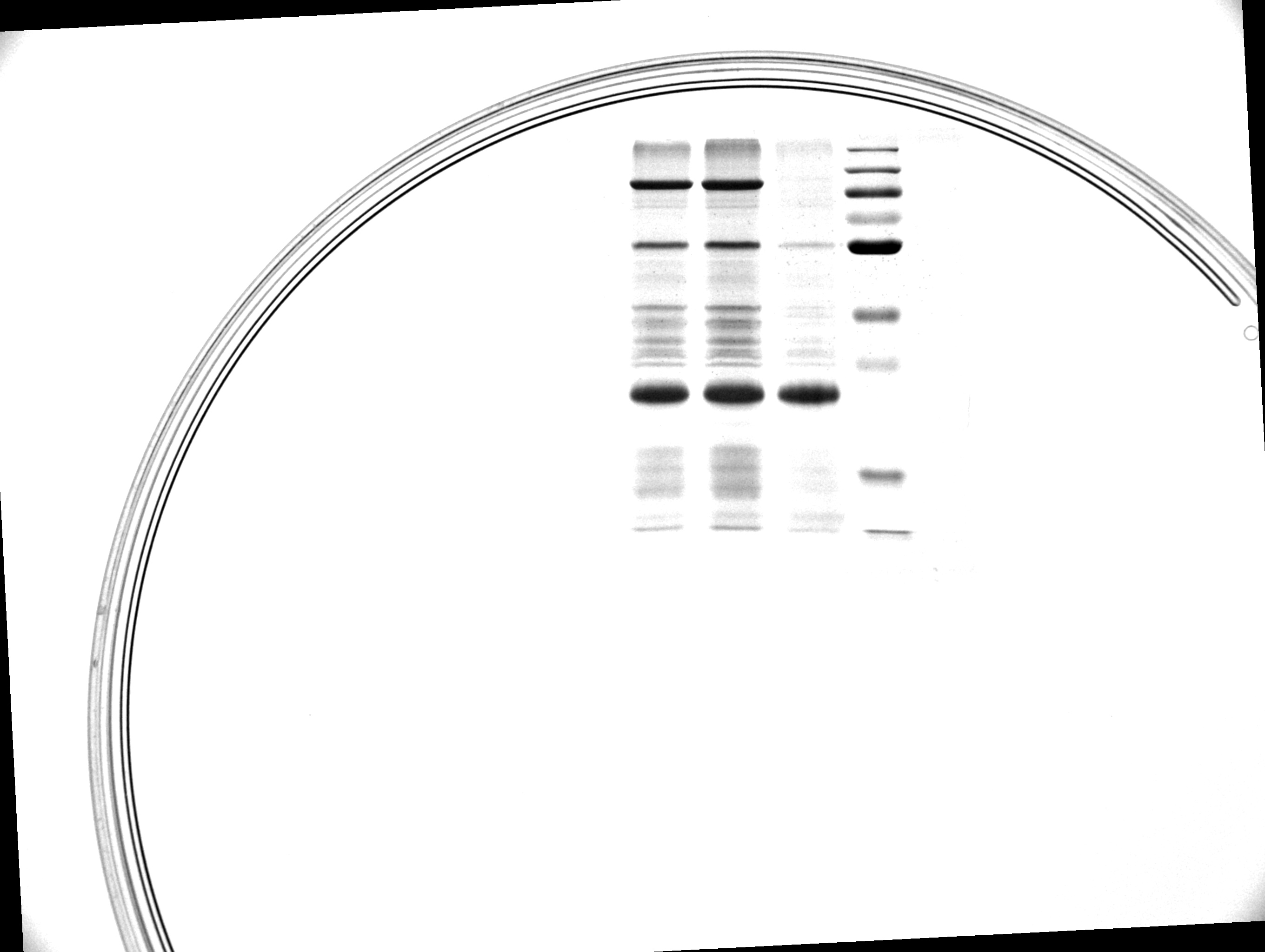

Supplement: Figure 4—source data 1. [file elife-91269-fig4-data1.zip › Figure_4-source_data_1/Figure_4-source_data_1_Figure_4B_IP Sliver staining.tif]

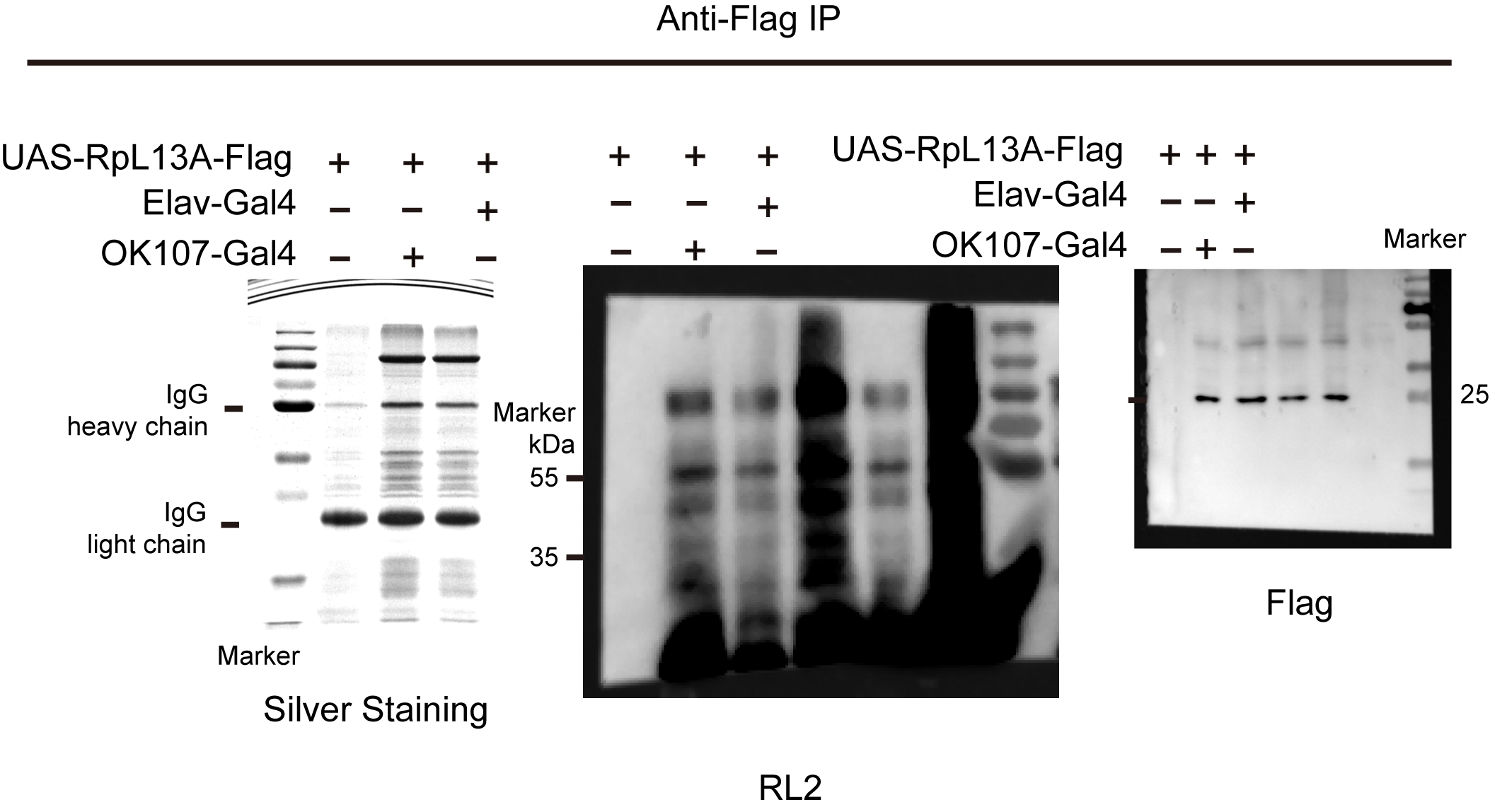

Supplement: Figure 4—source data 2. [file elife-91269-fig4-data2.zip › Figure_4-source_data_2/Figure_4-source_data_2-4B.tif]
